# Identification of copy neutral loss of heterozygosity on chromosomes 1p, 1q, and 6p among nonsyndromic cleft lip and/or without cleft palate with hypodontia

**DOI:** 10.1186/s12903-023-03464-3

**Published:** 2023-11-29

**Authors:** Norliana Ghazali, Normastura Abd Rahman, Thirumulu Ponnuraj Kannan, Azlina Ahmad, Sarina Sulong

**Affiliations:** 1 School of Dental Sciences, Universiti Sains Malaysia (USM), Health Campus, 16150 Kubang Kerian, Kelantan Malaysia; 2 Human Genome Centre, School of Medical Sciences, Universiti Sains Malaysia (USM), Health Campus, 16150 Kubang Kerian, Kelantan Malaysia

**Keywords:** Cleft lip, Cleft palate, Tooth loss, Loss of heterozygosity, Oligonucleotide array sequence analysis, Microsatellite repeats

## Abstract

**Background:**

Nonsyndromic cleft lip and/or without cleft palate (NSCL/P) with or without hypodontia is a common developmental aberration in humans and animals. This study aimed to identify the loss of heterozygosity (LOH) involved in hypodontia and NSCL/P pathogenesis.

**Methods:**

This is a cross-sectional study that conducted genome-wide copy number analysis using CytoScan 750K array on salivary samples from Malay subjects with NSCL/P with or without hypodontia aged 7–13 years. To confirm the significant results, simple logistic regression was employed to conduct statistical data analysis using SPSS software.

**Results:**

The results indicated the most common recurrent copy neutral LOH (cnLOH) observed at 1p33-1p32.3, 1q32.2-1q42.13 and 6p12.1-6p11.1 loci in 8 (13%), 4 (7%), and 3 (5%) of the NSCL/P subjects, respectively. The cnLOHs at 1p33-1p32.3 (D1S197), 1q32.2-1q42.13 (D1S160), and 6p12.1-6p11.1 (D1S1661) were identified observed in NSCL/P and noncleft children using microsatellite analysis markers as a validation analysis. The regions affected by the cnLOHs at 1p33-1p32.3, 1q32.2-1q42.13, and 6p12.1-6p11.1 loci contained selected genes, namely *FAF1*, *WNT3A* and *BMP5*, respectively. There was a significant association between the D1S197 (1p33-32.3) markers containing the FAF1 gene among NSCL/P subjects with or without hypodontia compared with the noncleft subjects (*p*-value = 0.023).

**Conclusion:**

The results supported the finding that the genetic aberration on 1p33-32.3 significantly contributed to the development of NSCL/P with or without hypodontia. These results have an exciting prospect in the promising field of individualized preventive oral health care.

## Introduction

Nonsyndromic cleft lip and/or without cleft palate (NSCL/P) with or without hypodontia is one of the most common congenital disabilities worldwide [1]. Cleft lip and/or cleft palate (CL/P) affects ~ 1 of every 700 live newborns, with considerable ethnic and geographical variations [2]. Previously, a study reported that 70% of orofacial cleft cases are nonsyndromic with multifactorial etiology, including genetic and environmental factors [3]. Patients with CL/P commonly present with a series of associated phenotypes such as including differences in the number, size, and shape of teeth compared with noncleft patients [4]. Tooth agenesis, widely known as hypodontia or congenital missing teeth, is the most regularly discerned developmental aberration of human dentition [5]. Hypodontia refers to the developmental failure of six or fewer teeth [6]. It is the most common dental anomaly in the Brazilian CL/P population, followed by impacted teeth, supernumerary teeth, and microdontia [7]. At the embryological stage, shared biological mechanisms contribute to the development of teeth, lips, and palates [8]. Therefore, we hypothesized that the genes controlling tooth formation are closely related to those contributing to the cleft formation. Previously, a significant association between markers in interferon regulatory factor 6 (IRF6), Wnt family member 3 A (WNT3A), and bone morphogenetic protein 5 (BMP5) genes among orofacial cleft and tooth agenesis was found; however, variants of Fas-associated factor 1 (FAF1) and grainyhead-like transcription factor 3 (GRHL3) genes contributed only to craniofacial development [9–12]. The IRF6 gene encodes a transcription factor that contributes to the formation of the oral ectoderm, periderm, and lip as well as the regulation of palatal shelf migration, adhesion, and fusion [13]. Mutation in IRF6 has been observed in 70% of families presenting with a combination of congenital lower lip pits, cleft palate alone or with cleft lip in some cases, and hypodontia [14]. The mutation in both GRHL3 and IRF6 genes exhibited the abnormal oral periderm and lead to nearly identical phenotypes of orofacial [15]. The FAF1 gene functions as a regulator of cranial neural crest (CNC) differentiation and influences the development of cleft palate in humans, as observed in zebrafish [16]. Variations in WNT3 interact with other Wnt genes expressed in neural crest cells that may disturb formation and fusion of the processes and influence tending to NSCL/P [17]. A previous study demonstrated that canonical Wnt signaling, which is expressed in the dental epithelium and mesenchyme, may influence tooth formation, and the disruption of this signaling may arrest tooth morphogenesis [18]. BMP signaling is also crucial for tooth development as it regulates early tooth morphogenesis and palatogenesis processes [19]. In the literature review, nine genomic loci were identified, including 1q21-q25, 1q32, 2q31.2-q33.2, 4p16.3, 8q24, and 16q22, leading to the occurrence of CL/P and hypodontia [20].

Various genetic approaches have been employed to identify genes and genomic loci that contribute to the development of CL/P and hypodontia, including animal model expression studies, candidate gene sequencing, and genome-wide association studies. Recently, the role of chromosomal deformities in genetic defects and diseases has been investigated [21]. Copy number variations (CNVs) and loss of heterozygosity (LOH) alter gene expression, disrupt gene sequences, and change gene dosage [22]. To date, the co-occurrence of hypodontia and NSCL/P remain poorly understood. The deletion of 1q21-q25 encompassed 702 genes, including protein-coding genes, noncoding genes, miRNAs, and long noncoding RNAs, detected in syndromic orofacial cleft and tooth agenesis [21]. However, investigations on CNVs and genes related to NSCL/P with hypodontia are limited. The causative genes and genomic loci of syndromic CL/P with hypodontia may also play a role in the development of hypodontia in NSCL/P. Therefore, a chromosomal microarray analysis was conducted using the CytoScan 750K array to determine the contribution of genetic aberrations to the development of NSCL/P with hypodontia. This study focused on the identification of significant LOH and genes contributing to the development of NSCL/P in hypodontia. Validation analysis was then conducted on the significant gene in LOH using microsatellite markers.

## Materials and methods

### Study design

This comparative cross-sectional study included 81 individuals (61 NSCL/P cases and 20 noncleft cases) aged 7–13 years. Patient samples were obtained from one of the tertiary hospitals in northeastern Malaysia between 2016 and 2018. The inclusion criteria were children with NSCL/P aged 7–13 years while, those with a cleft palate and patients with other syndromes such as ectodermal dysplasia or Axenfeld–Rieger syndrome were excluded. The comparison group consisted of noncleft children with their age ranging between 7 and 13 years. Based on examination and history taking, healthy children with no orofacial cleft deformities and no family history of NSCL/P were also included. However, those who had undergone orthodontic treatment were excluded from the study. This study has a sample population and design similar to those of the previous study [23]. The sample size for genetic abnormalities was calculated using the single proportion formula based on the prevalence of genetic aberrations [24]. This study recruited 61 NSCL/P and 20 noncleft children for genetic alteration analysis. The sample size was used based on the study time and financial limitations. Dental panoramic tomography was performed to identify and confirm the number and morphology of the teeth. This study was approved by the Human Research Ethics Committee of Universiti Sains Malaysia (USM) (Approval number: USM/JPEPeM/140357). The sample collections and all experiments were performed in accordance with the guidelines and regulations of the Human Ethics Committee and the Research Committee of USM. The individuals and their guardians had received an explanation of the complete research protocol and signed the informed consent before study enrolment.

### DNA extraction

Saliva samples were collected from each patient and stored in sterile 50 mL conical tubes. Genomic DNA was extracted from the samples according to the GeneAll Blood SV Mini Kit manual (General Biosystems, Seoul, South Korea), which included lysis, binding, washing and elution.

### CytoScan 750K array analysis and loss of heterozygosity analysis

Genome-Wide Human CytoScan 750K Array (Affymetrix, CA, United States) was used to analyze genomic alterations according to the manufacturer’s protocol. Genomic DNA (250 ng) was digested with NspI for 2 h at 37 °C. The digested DNA was ligated with adaptors at 16^o^C and 70 °C for 3 h and 20 min, respectively. Amplicon was generated by performing polymerase chain reaction (PCR) on the adaptor-ligated DNA using reagents provided by Affymetrix with the following settings: 94 °C for 3 min, then 30 cycles of 94 °C for 30 s, 60 °C for 45 s, and 65 °C for 1 min, followed by extension at 68 °C for 7 min. The PCR products were then purified and fragmented with DNase 1 for 35 and 15 min at 37 °C and 95 °C, respectively. Subsequently, the fragmented DNA was labeled with biotinylated for 4 h at 37 °C. Fragments of DNA (200 µg) were hybridized with pre-equilibrated Affymetrix Chip CytoScan 750K at 50 °C for 18 h. The arrays were then washed and stained using GeneChip Fluidics Station 450 and Affymetrix GeneChip Command Console Software, followed by data analysis using Affymetrix Chromosome Analysis Suite (ChAS) software.

### Loss of heterozygosity analysis

CytoScan750K array technology was used to define allelic imbalances in LOH. LOHs were recognized accordingly using the ChAS software based on the graph of the allelic difference produced. Areas of LOHs are represented by the absence of plots at the value of 0. Copy number status was checked to categorize the allelic imbalances into conventional LOH (CN = 1), copy-neutral LOH (cnLOH; CN = 2), or LOH with complimentary copy number gains (CN ≥ 3).

### Microsatellite marker analysis

Microsatellite DNA loci are valuable markers for LOH detection. The significance of LOH results obtained from the CytoScan 750K array were undergo validated analysis using microsatellite marker analysis. LOH is described as the loss of one allele at a heterozygous location, causing the cell to have only one version of a gene. In this study, the results of the CytoScan 750K array indicated that genomic areas such as FAF1 (1p33-32.3), WNT3A (1q32.2-42.13), and BMP5 (6p12.1-11.1) were analyzed for cnLOH. Three informative microsatellite markers (D1S1601, D1SI97, and D6S1661) located on chromosomes 1p33-32.4, 1q32.2-42.13, and 6p12.1-11.1 were used in this study. Primers were designed and one primer from each pair was end labeled with a fluorescent dye. Table 1 presents the primer sequences and type of microsatellite repeats. PCR amplification was performed in a total reaction volume of 25 µL. Each PCR mixture contained 100 ng of sample DNA, PCR Master Mix (2X), 10-µM forward and reverse primers, and nuclease-free water (Promega Corporation, USA). The cycling conditions were initial denaturation at 95 °C for 5 min, followed by 34 cycles of denaturation at 94 °C for 30 s, annealing at 58 °C for 20 s, extension at 72 °C for 30 s, and then final extension at 72 °C for 5 min.

**Table 1:** Microsatellite primers on chromosome 1q32.4-1q42.13, 1p33-1p32.3 and 6p12.1-6p11.1

Markers	Region	Primer sequences (5’-3’)
D1S160 (*WNT3A*)	1q32.4-1q42.13	Forward : TTCCTGTTTGATGTTCAAAGG Reverse : CAGAGAGCATCTGGGGACTA
D1S197 (*FAF1*)	1p33-1p32.3	Forward : TCCTTTTCTCATGTCCCTCCTCC Reverse : GATGACAAAGAGAGCAAGGATCC
D6S1661 (*BMP5*)	6p12.1-6p11.1	Forward : GGGCCTTTCAAAAATGGA Reverse : TTGCAAAGACTGACATGAATTG

### Genetic fragment analysis

Each PCR product (1 µL) was mixed with 0.25 µL of GeneScan 400HD ROX Size Standard (Applied Biosystems, USA). Each sample was denatured at 95 °C for 5 min and then chilled on ice for 10 min. Capillary electrophoresis analysis was conducted on an ABI 3100 Genetic Analyzer and data were analyzed using GeneScan Analysis 3.7 software. The results could be classified as loss of one allele or LOH, appearance of both alleles (retention of heterozygosity), or involvement of only one allele (noninformative [NI]).

### Statistical analysis

Statistical analysis was conducted using SPSS version 26.0. LOH frequency in selected markers was compared between NSCL/P and noncleft subjects with or without hypodontia using simple logistic regression analysis to determine significant chromosome alterations.

## Results

### Genome-wide assessment of the loss of heterozygosity in nonsyndromic cleft lip and/or without cleft palate (NSCL/P) and noncleft with or without hypodontia

**Fig. 1:**
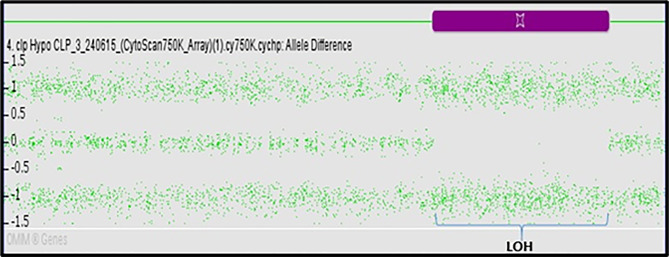
Identification of LOHs based on the allele difference graph difference graph generated from the chromosome analysis suite (ChAS) software. Regions of LOHs denoted by the absence of plots at the value of 0

This study included 81 subjects (61 NSCL/P and 20 noncleft cases) aged between 7 and 13 years. Their salivary samples were subjected to genetic aberration assay using the CytoScan 750K array. The mean (standard deviation) ages of the NSCL/P and noncleft subjects were 10.7 ± 1.83 and 9.9 ± 2.1, respectively. Besides CNVs, genome-wide array technology was also used to describe allelic imbalances in LOH. LOHs were recognized accordingly using the ChAS software based on the graph of the allelic difference produced (Fig. 1). The regions of LOHs are denoted by the absence of plots at the value of 0. Copy number status was checked to classify the allelic imbalances into conventional LOHs (CN = 1), cnLOH (CN = 2), or LOHs with complimentary copy number gains (CN ≥ 3).

A total of 430 LOHs in 303 genomic loci were identified in NSCL/P and noncleft subjects with or without hypodontia. Of these LOHs, 121 (30%) exhibited allelic imbalances with copy number losses, and 309 (70%) were LOHs without change in the cnLOH. Of all the allelic imbalances, the most common recurrent cnLOH was observed at 1p33-1p32.3, 1q32.2-1q42.13, and 6p12.1-6p11.1 loci in 8 (13%), 4 (7%), and 3 (5%) NSCL/P cases, respectively. The percentages of cnLOH among NSCL/P subjects with or without hypodontia were 18% (17p11.2), 18% (20q11.21-20q11.23), and 12% (22q13.1-22q13.2) (Table 2). On the other hand, as shown in Table 3 the highest percentages of cnLOH among noncleft subjects with hypodontia were 18% (20q11.21-20q11.23), 12% (22q13.1-22q13.2) and 11% (Xp11.23-Xp11.22).The percentage of LOH was determined by dividing the length of the chromosome affected by LOH by the length of the entire chromosome arm. The most frequently altered regions among the noncleft subjects with hypodontia were 1p33-1p32.3 (10%) and 6p12.1-6p11.1 (10%), both of which exhibited no copy number alteration (Table 3).

**Table 2:** Frequency and percentage of LOH in more than one patient among 61 NSCL/P with or without hypodontia

LOH Status	Chr	Cytoband start	Cytoband end	Size (Mb)	No of sample	Frequency of LOH (%)	Percentage of LOH (%)
cnLOH	1	1p33	1p32.3	5.494	8	13	4
	1	1p36.11	1p35.3	4.481	2	3	3
	1	1q32.2	1q42.13	20.469	4	7	16
	2	2p23.3	2p23.2	3.488	3	5	4
	2	2p12	2p11.2	5.735	3	5	6
	2	2q32.1	2q32.2	4.747	3	5	3
	2	2q11.1	2q11.2	6.711	2	3	5
	4	4q32.1	4q32.2	7.812	2	3	6
	6	6p12.1	6p11.1	3.993	3	5	7
	7	7q31.33	7q32.2	3.622	2	3	4
	7	7q11.21	7q32.2	4.613	3	5	5
	10	10p12.31	10p12.1	3.060	2	3	10
	10	10q22.1	10q22.2	3.398	2	3	4
	11	11p11.2	11p11.12	6.057	2	3	6
	11	11q11	11q12.1	3.646	3	5	4
	12	12q21.32	12q21.33	3.557	2	3	4
	15	15q15.1	15q15.3	3.542	2	3	4
	17	17p11.2	17p11.2	4.635	2	3	18
	17	17q22	17q23.2	3.9006	2	3	6
	20	20q11.21	20q11.23	6.572	2	3	18
	22	22q13.1	22q13.2	4.111	2	3	12
LOH	X	Xq11.1	Xq13.1	6.128	8	13	6

**Table 3:** Frequency and percentage of LOH among non-cleft with or without hypodontia (n = 20)

LOH status	Chr	Cytoband start	Cytoband end	Size (Mb)	No of sample	Frequency of LOH (%)	Percentage of LOH (%)
cnLOH	1	1p33	1p32.3	5.496	2	10	4
	1	1p36.11	1p35.3	3.335	2	10	3
	1	2q32.2	1q42.13	20.469	1	5	3
	1	2q25.2	2q25.2	3.396	2	10	3
	2	2q11.1	2q11.2	5.856	2	10	4
	6	6p12.1	6p11.1	5.523	2	10	4
	6	6p22.2	6p21.33	4.907	2	10	8
	11	11p11.2	11p11.12	4.167	2	10	8
	14	14q23.1	14q23.2	3.542	2	10	4
	20	20q11.21	20q11.23	6.572	2	10	18
	22	22q13.1	22q13.2	4.111	2	10	12
LOH	X	Xp11.23	Xp11.22	7.044	2	10	11
	X	Xq11.1	Xq13.1	7.156	6	30	8

**Fig. 2:**
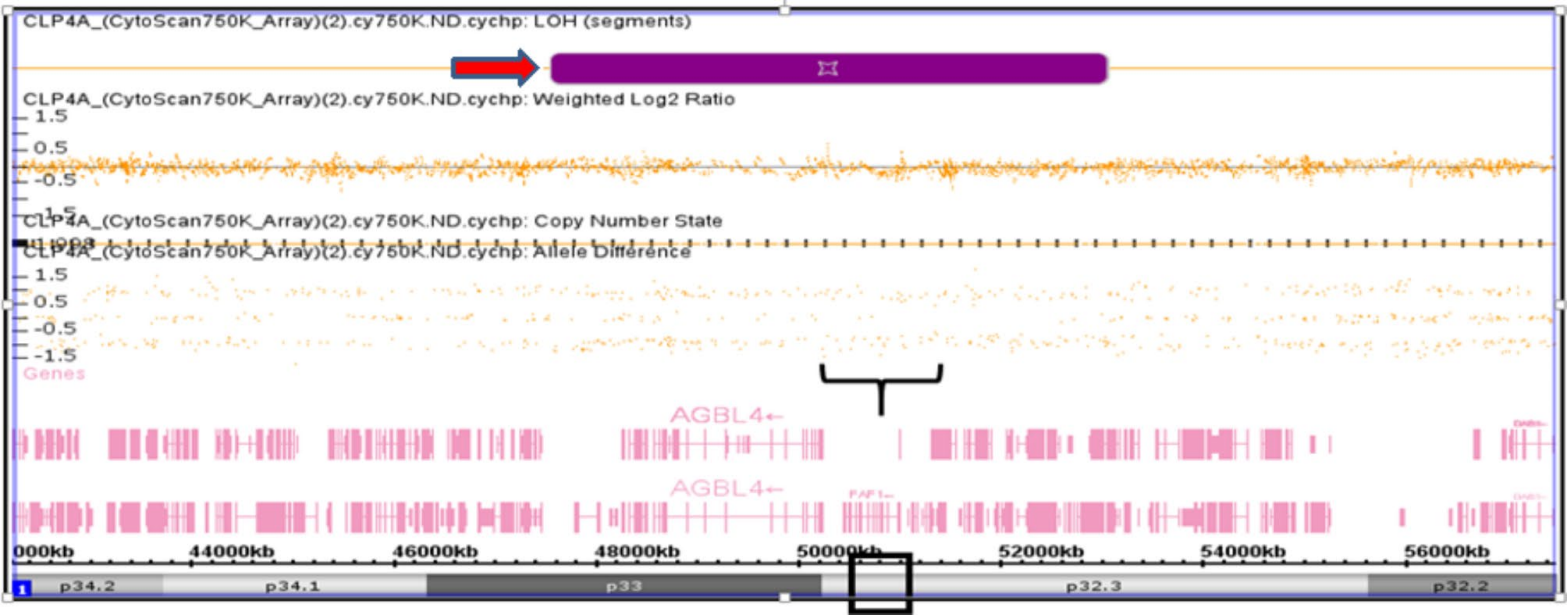
SNP-Array (Affymetrix CytoScan 750K Array) report case CLP4. No CNVs were detected but LOH was observed on short arm of chromosome 1 (1p33-1p32.3, showed as red arrowhead). Black box showed the LOH region at FAF1 gene location.

**Fig. 3:**
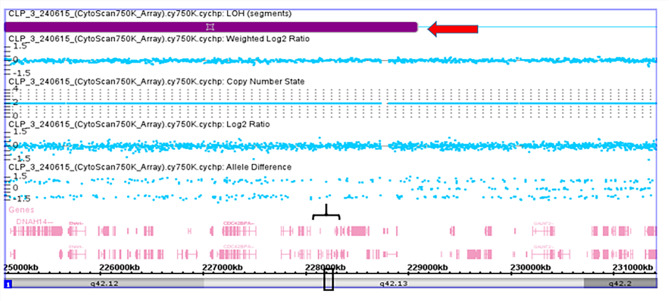
SNP-Array (Affymetrix CytoScan 750K Array) report case CLP3. No CNVs were detected but loss of LOH was observed on long arm of chromosome 1 (1q32.2-1q42.13), showed as red arrowhead). Black box showed the LOH region at WNT3A gene location.

**Fig. 4:**
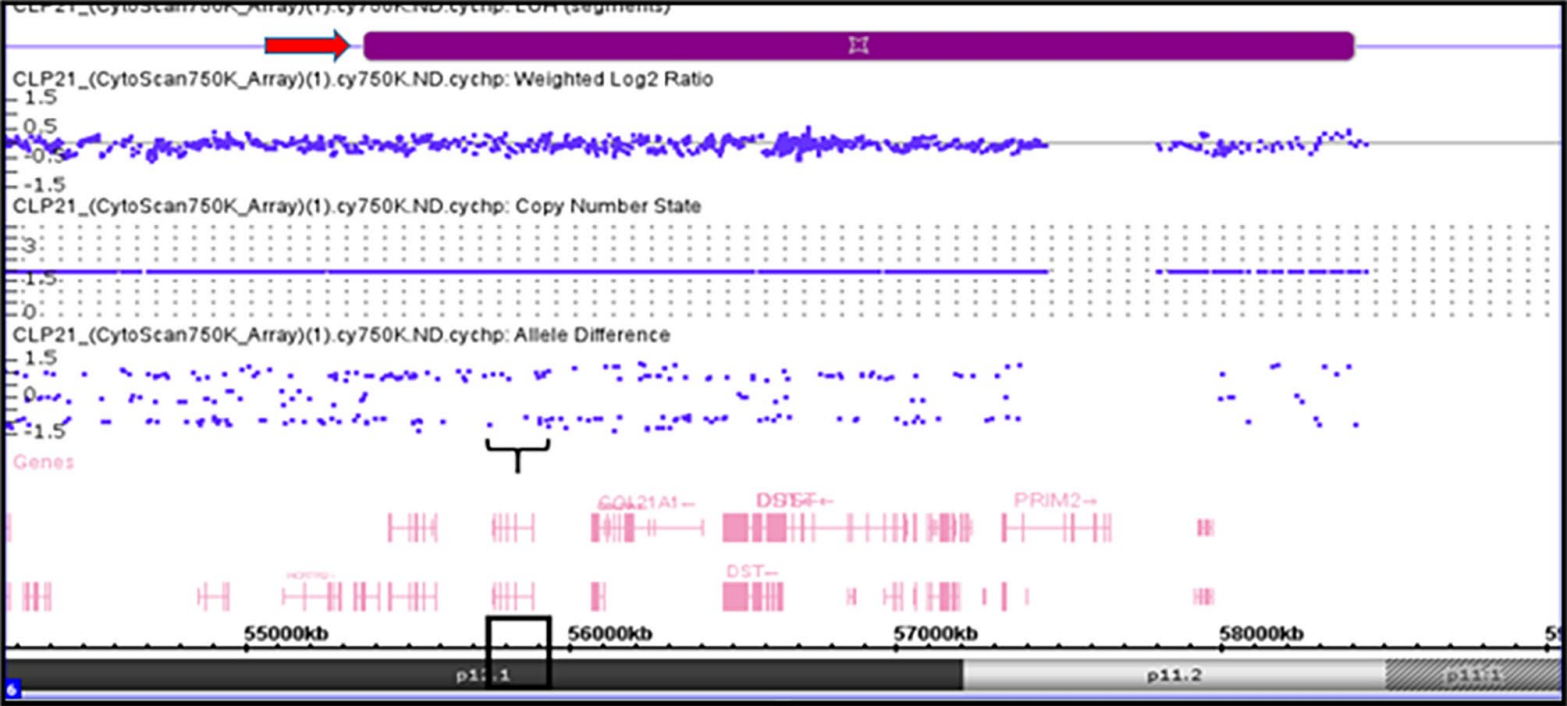
SNP-Array (Affymetrix CytoScan 750 K Array) report case CLP21. No CNVs were detected but LOH on short arm of chromosome 1 (6p12.1-6p11.1) was observed showed as red arrowhead. Black box showed the LOH region at BMP5 gene location.

Figure 2 presents a detailed view of the LOH based on the allele difference graph generated from the ChAS software for the case of CLP4. Genomic annotation revealed that the affected region of the 1p33-1p32.3 locus contained 14 genes; the following genes were observed in all the affected subjects based on the SNP Array (Affymetrix CytoScan 750K Array) report of the CLP4 case: *AGBL4*, *BEND5*, *LOC101929721*, *AGBL4-IT1*, *ELAVL4*, *DMRTA2*, and *FAF1*. Figure 3 presents a reported case (CLP3) based on the SNP Array (Affymetrix CytoScan 750K Array). No CNVs were detected, but LOH on the long arm of chromosome 1 (1q32.2-1q42.13) is called cnLOH. The region affected by cnLOH contains many genes, including *WNT3*. Figure 4 presents the SNP Array (Affymetrix CytoScan 750K Array) report of the CLP21 case. No CNVs were detected, but LOH was detected on the short arm (p) of chromosome 6. Furthermore, 18 genes were mapped to the LOH region of 6p12.1-6p11: *GFRAL*, *HMGCLL1*, *BMP5*, *COL21A1*, *DST*, *DST-AS1*, *BEND6*, *KIAA1586*, *ZNF451*, *ZNF451-AS1*, *BAG2*, *RAB23*, *LOC100506188*, *PRIM2*, *MIR548U*, *GUSBP4*, *LINC00680-GUSBP4*, and *LINC00680*.

### Validation of the loss of heterozygosity through microsatellite marker analysis

A total of 20 NSCL/P and noncleft subjects with hypodontia were analyzed using the D1S197, D1S160, and D1S1661 markers to validate LOH on chromosomes 1p33-1p32.3, 1q32.2-1q42.13, and 6p12.1-6p11.1, respectively. Each sample was matched with a normal (noncleft) sample, and each marker was interpreted based on the presence or absence of the correct allele (peak size). The presentation of microsatellite markers with two distinct alleles in the normal control or the NSCL/P and noncleft subjects with or without hypodontia is considered an informative result, whereas the presence of only one allele in both patients and normal control indicates an NI result. LOH is defined as the complete disappearance of one allelic peak from the patient’s DNA or the reduction of the signal intensity (allelic ratio of < 0.05) by < 50% in the patient’s DNA compared with the paired normal DNA pattern.

**Fig. 5:**
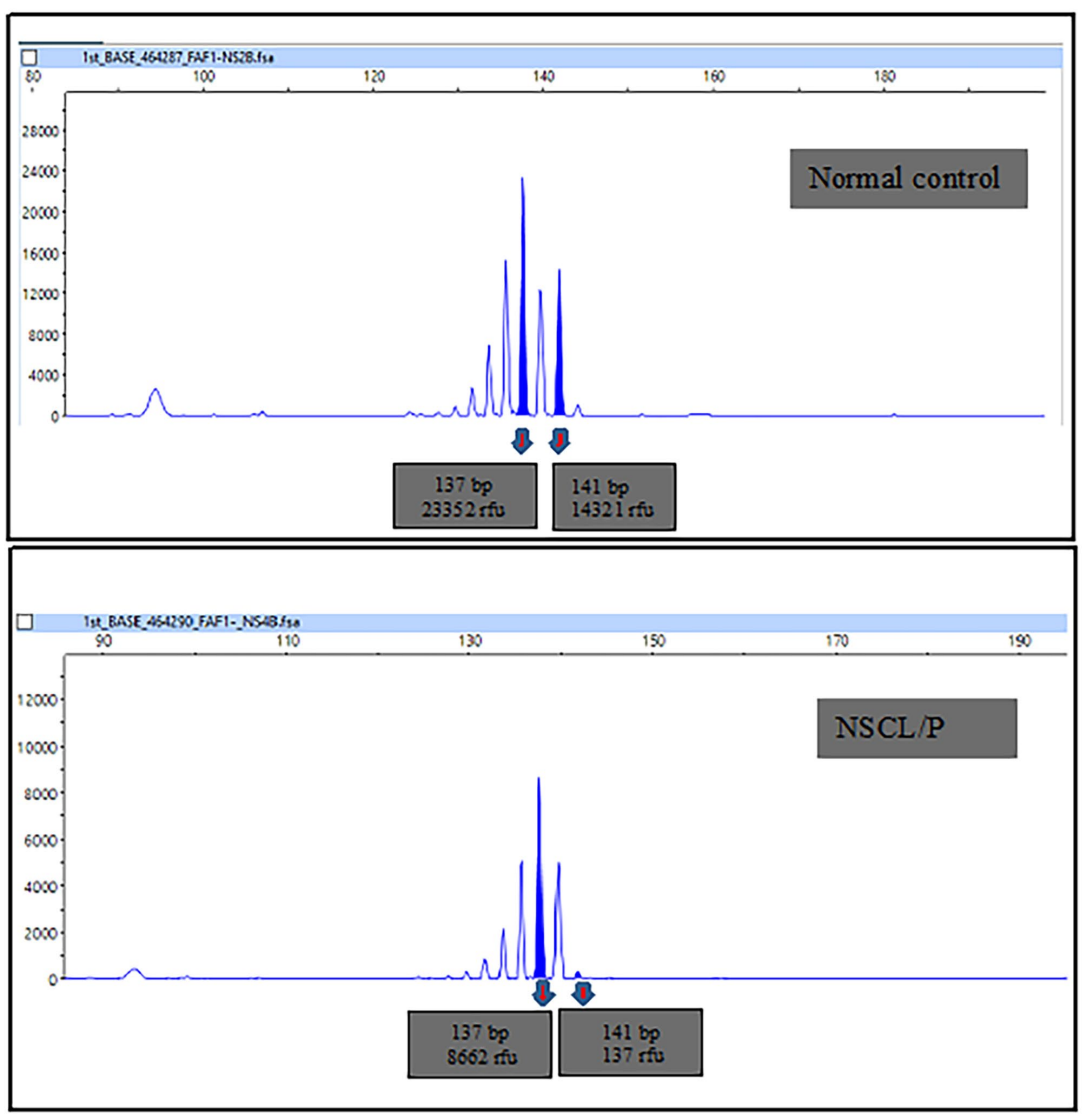
Representative picture of microsatellite analysis showing LOH at markers D1S197. In the normal control, the two allele of this microsatellite are 137 and 141 base pairs long. In NSCL/P subjects, loss of peaks at 141 bp indicated loss one allele and are scored as LOH

**Fig. 6:**
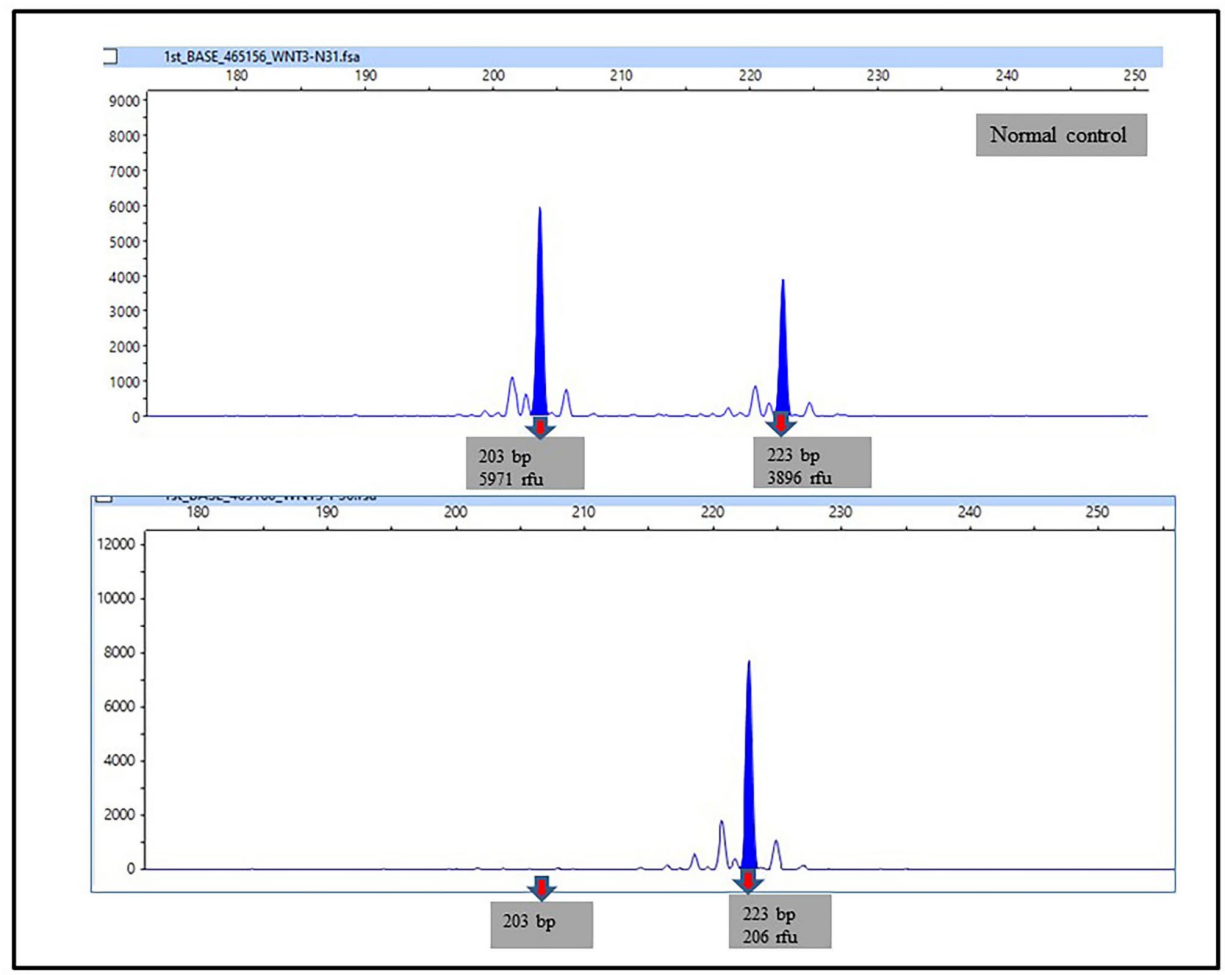
Representative picture of microsatellite analysis showing LOH at D1S160 markers. In the normal control, the two allele of this microsatelite are 203 and 223 base pairs long. In NSCL/P subjects, loss of peaks at 203 bp indicated loss one allele and are scored as LOH

Figure 5 presents a representative picture of microsatellite analysis showing LOH at the D1S197 marker. In the normal control, the two alleles of this microsatellite were 137 and 141 base pairs long. In the NSCL/P subjects, loss of peaks at 141 bp indicated the loss of one allele and was scored as LOH. Eight NSCL/P and two noncleft patients with a high probability of LOH at 1p33-1p32.3 based on the CytoScan 750K array demonstrated LOH at the D1S197 microsatellite marker (Table 4). The results of the microsatellite analysis confirmed the chromosomal regions with high probability of LOH detected by the CytoScan 750K array. All subjects with no LOH based on the CytoScan 750K array had similar negative results in microsatellite marker analysis. Figure 6 presents the microsatellite marker analysis for the NSCL/P children on chromosome 1q32.2-1q42.13, indicating that the allelic peak completely disappeared. Additionally, two of the noncleft subjects were interpreted as having a total loss of 203-bp allelic peak.

**Table 4:** Association between LOH of selected markers and Hypodontia in non- syndromic Cleft Lip and/or Palate Children

Variables	NSCL/P With or without Hypodontia Freq (%)	Non-cleft with or without Hypodontia Freq (%)	Crude OR	LR Statistic	p-value
			OR 95% CI		
D1S197 (1p33–32.3)					
LOH	8 (80)	2 (20)	0.063 0.007–0.559	7.2	0.023
No LOH	2 (20)	8 (80)			
**D1S160 (1q32.2-42.13)**					
LOH	4 (40)	1 (10)	0.167 0.15–1.879	2.4	0.303
No LOH	6 (60)	9 (90)			
**D6S1661 (6p12.1-11.1)**					
LOH	3 (30)	2 (20)	0.583 0.075–4.562	0.267	1.00
No LOH	7 (70)	8 (80)			

**Fig. 7:**
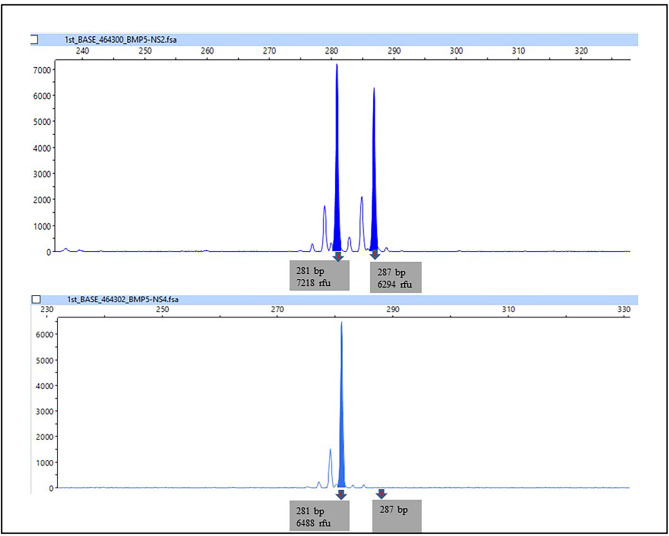
Representative picture of microsatellite analysis showing LOH at markers D6S1661. In the normal control, the two allele of this microsatellite are 281 and 287 base pairs long. In NSCL/P subjects, loss of peaks at 141 bp indicated loss one allele and are scored as LOH.

Figure 7 presents the representative of microsatellite marker analysis for the NSCL/P subject on chromosome 6p12.1-6p11.1, indicating that the allelic peak completely disappeared. In addition, two of the noncleft subjects were interpreted as having a total loss of 281-bp allelic peak. The results from the microsatellite analysis confirmed the chromosomal regions with high probability of LOH detected by the CytoScan 750K array. All subjects with no LOH based on CytoScan 750K had similar negative results in microsatellite marker analysis. The representative picture of microsatellite analysis showing LOH at the D6S1661 marker. In the normal control, the two alleles of this microsatellite were 281 and 287 base pairs long. In the NSCL/P subjects, loss of peaks at 287 bp indicated loss of one allele and was considered as LOH. Chi-squared tests were conducted to determine the significance of the association of the LOH of the selected markers with NSCL/P and noncleft with or without hypodontia. No significant association was observed between the LOH of D1S160 (1q32.2-42.13) and D6S1661 (6p12.1-11.1) and NSCL/P and noncleft with or without hypodontia. However, there was a significant association between the D1S197 (1p36.33-32.3) marker and the *p*-value of 0.023 (Table 4).

## Discussion

### Characterization of the loss of heterozygosity among in nonsyndromic cleft lip and/or without cleft palate (NSCL/P) and noncleft with or without hypodontia

A total of 303 genomic loci, including 430 LOHs, were detected in NSCL/P and noncleft subjects with or without hypodontia. The results indicated that 30% of these aberrations exhibited allelic imbalances with copy number changes, whereas 70% were LOHs without change in the cnLOH. These results suggest that cnLOH is higher than LOHs with copy number changes. cnLOH, also known as uniparental disomy (UPD), occurs when a maternal (unimaternal) or paternal (unipaternal) chromosome or chromosomal region is duplicated and the other allele is lost [25]. In this study, simultaneous analysis of the DNA copy number status and allele zygosity enabled the categorization of the LOH into classical LOHs, cnLOHs, or LOHs with accompanying copy number gain. This study demonstrated that most (70.0%) of LOHs in NSCL/P existed in the form of cnLOHs. Although data regarding the prevalence of cnLOHs in NSCL/P are limited, a previous study has demonstrated that cnLOHs might influence gene expression and lead to the disruption of embryonic development [26]. Duplication of an activating mutation in an oncogene, deletion or loss of function of a tumor suppressor gene, and duplication or deletion of a methylation allele that regulates gene expression can all result from a cnLOH [27]. LOHs at Xp22.33-Xp11.1 and Xq11.1-Xq28 often recurred, which was not surprising given the prevalence of LOHs at the X chromosome in diseases such as cancer. As males have only one copy of the X chromosome and females have inactivated X chromosomes, this can be considered as the first strike of Knudson’s two-hit hypothesis. The cnLOH recurred in chromosomes 1p33-1p32.3, 1p32.2-1q42.13, and 6p12.1-6p11.1 in NSCL/P subjects with or without cleft palate, whereas 1p33-1p32.2 and 6p12.1-6p11.1 were commonly detected in noncleft subjects without hypodontia.

Microsatellites, also known as short tandem repeats, is a genetic variant unique to specific chromosomes and sites that can be used to validate LOH and cnLOH. Due to their rapid mutation, large number of alleles, and frequency in genomic DNA, microsatellites are particularly important molecular markers in population genetics, genome mapping, taxonomic study, linkage analysis, genetic fingerprinting, and diversification [28]. The cnLOH, also known as UPD, causes LOH by duplicating unipaternal chromosomes or chromosomal regions and simultaneously losing the other allele. LOH validation was performed using microsatellite marker (D1S197) analysis to confirm 1p33-1p32.3 regions with a high risk of LOH detected by the CytoScan 750K array. The present study demonstrated a significant cnLOH at the D1S197 marker on chromosome 1p33-1p32.3 encompassing the FAF1 gene. *FAF1* encodes the *FAF1* protein that binds to the FAS antigen and initiates apoptosis [29]. *FAF1* has been demonstrated to play a critical role in normal neuronal cell survival and development, whereas *FAF1* downregulation has been linked to numerous aspects of carcinogenesis [29].

An ongoing research is expected to shed light on the role of *FAF1* in signaling pathways critical for normal development and cancer. The loss of peaks at 141 bp indicated the loss of one allele in the sample with LOH on 1p33-1p32.3 and was evaluated as cnLOH. Most subjects required two peaks representing 137 and 141 base pairs. The FAF1 gene was selected from the LOH area as it regulates CNC. CNC is a migrating cell population that forms most craniofacial bones [11]. Tooth and palate growth requires a series of interactions between the pharyngeal ectoderm and CNC-derived mesenchyme [30]. *FAF1* has been found to play an important role in normal neuronal cell survival and proliferation, whereas *FAF1* downregulation may play a role in cancer through a variety of mechanisms [31]. *FAF1* blocks Wnt signaling by increasing catenin breakdown [32]. Cell proliferation, differentiation, organogenesis, tissue regeneration, and cancer are all affected by Wnt signaling [33]. A previous study suggested that the Wnt signaling system affects palate and tooth growth [34]. Activation of the odontogenic mesenchyme during the early development of teeth, lips, and palates and cancer is dependent on Wnt/beta-catenin signaling [35, 36]. These results further support the finding that *FAF1* interacts with the transforming growth factor (TGF)-*β* type II receptor, inhibiting *TGF* signaling and thus suppressing epithelial-to-mesenchymal transition (EMT) [37]. EMT is required for the fusion of palate shelves. During the fusion of palate shelves, TGF signals have stimulated both medial edge epithelium and apoptosis [38].

The D1S160 marker was used to confirm LOH on chromosome 1q32.2-1q42.13, which includes the WNT3A gene in 20 NSCL/P and noncleft individuals with or without hypodontia. The CytoScan 750K assay indicated that chromosome 1q32.2-1q42.13 was selected as one region of interest with LOH related to NSCL/P with hypodontia. This LOH region was observed in five NSCL/P samples. This modification does not affect the copy number, which is known as cnLOH. LOH validation was performed through microsatellite marker (D1S160) analysis to confirm the 1q32.2-1q42.13 regions with high probability of LOH detected by CytoScan 750K array. The loss of peaks at 203 bp indicated the loss of one allele and was evaluated as LOH in the sample with LOH on 1q32.2-1q42.13. The typical subjects must have two peaks representing 203 and 223 base pairs. The WNT3A gene belongs to the Wnt gene family, which plays an important role in prenatal development. The WNTA genes code for proteins involved in the chemical signaling pathways of the body. These pathways control the activity of certain genes and cell interactions throughout embryonic development [39]. In the mouse embryo, WNT signaling is necessary for proper head and face development, and it is implicated in many aspects of craniofacial development, including axis formation, CNC cell survival, and brain patterning [40]. This study discovered recurrent cnLOH in NSCL/P, which contributed to the WNT3A gene, at 1q32.2-1q42.13. When a person inherits two copies of a chromosome or part of a chromosome from one parent but none from the other, it is known as cnLOH or UPD. Wnt genes control midface development and upper lip fusion, according to a previous study. As a result, it could play a role in NSCL/P [40]. Wnt also contributes to tooth development. Based on the results of this study, we can assume that *WNT3A* also contributed to the formation of NSCL/P and hypodontia. This statement is supported by the findings of functional studies and experimental examination of relevant animal models that validate the impact of the WNT signaling system on tooth development and regulation of adult tooth homeostasis [41].

The 6p12.1-6p11.1 locus represented an attractive genomic region in which cnLOHs occurred. cnLOH at 6p12.1-6p11.1 recurred at frequencies of 3% and 2% in NSCL/P subjects with hypodontia and noncleft subjects with hypodontia, respectively. This finding suggests that genes residing at this genomic locus are involved in the regulation of programmed cell death, migration of epithelial cells to the oral or nasal side of the palate, and EMT and disturbances during the early stages of tooth development. Among the specimens examined in this study, LOHs at 6p12-6p11.1 were found to affect eight genes. Of these, *BMP5* has been reported to contribute to palatogenesis and odontogenesis [30]. This gene encodes a protein that is a member of the TGF superfamily. The potential of BMPs to stimulate bone and cartilage formation is well established. *BMP5* may play a role in the development of certain malignancies. The *BMP* signaling pathway regulates various developmental processes such as cell proliferation, apoptosis, differentiation, and morphogenesis [42]. This suggests that LOHs at 6p12-6p11.1 facilitate *BMP5* function through the *TGF-β* pathways. This is because the *TGF-β* superfamily includes activin, *TGF-β*, and *BMP* cytokines. Chi-squared tests were conducted to determine the significance of the association of the LOH of selected markers with NSCL/P and noncleft with or without hypodontia. The LOH of D1S160 (1q32.2-42.13) and D6S1661 (6p12.1-11.1) was not found to be associated with NSCL/P and noncleft palate with or without cleft palate. However, there was a significant association between D1S197 (1p36.33-32.3) markers and a *p*-value of 0.023.

This study has few limitations that need to be acknowledged for future work. First, the small sample size could limit the statistical power of the analysis. Therefore, future studies are recommended to use a larger number of samples and, whenever possible, include an independent cohort of cases and control from different tertiary centers for the result validation. Additionally, discussions should be made on the mechanism underlying the risk association of genes in the region contributing to LOH based on available literature. Future studies should include appropriate experimental works such as chromatin immunoprecipitation assay to explore the interactions between genes and proteins to uncover the actual mechanism behind the associations. Additionally, for the gene expression study, analysis at the protein level could be considered in the future because genes would eventually be translated into proteins, and posttranslationally modified proteins are the actual molecules of the cells. Future studies should also try establishing a correlation between genomic abnormalities (copy number alterations and LOHs) and gene expression changes in NSCL/P and hypodontia to provide novel insights into the mechanism of palatogenesis and odontogenesis.

## Conclusion

Herein, a genome-wide analysis was conducted to investigate LOHs in NSCL/P patients and uncover probable NSCL/P with hypodontia-related genes. We found three recurrent LOHs in the chromosome at 1p33-1p32.3, 1p32.2-1q42.13, and 6p12.1-6p11.1 loci in NSCL/P subjects with or without cleft palate, whereas 1p33-1p32.2 and 6p12.1-6p11.1 were commonly detected in noncleft subjects without hypodontia. Our findings add to the pool of potential causal markers for NSCL/P and hypodontia for future genetic investigations. Human mutational analysis and animal model research are warranted to confirm the role of the identified putative causal genes of NSCL/P and hypodontia.

## Data Availability

The data that support the findings of this study are available from the corresponding author upon reasonable request.

## Data Availability

The datasets generated and analysed during the current study are available in the online repositories in ClinVar database with Accession id as following: VCV001809510.1(*BMP5*;https://www.ncbi.nlm.nih.gov/clinvar/variation/1809510/), VCV001809607.1 (*WNT3A*;https://www.ncbi.nlm.nih.gov/clinvar/variation/1809607/), VCV001809606.1 (*FAF1*); https://www.ncbi.nlm.nih.gov/clinvar/variation/1809606/.

## Abbreviations

NSCL/P: Non-syndromic cleft lip and/or without cleft palate
LOH: Loss of heterozygosity
cnLOH: Copy neutral loss of heterozygosity
CL/P: Cleft lip with or without cleft palate
*IRF6*: Interferon regulatory factor 6
*WNT3A*: Wnt family member 3 A
*BMP5*: Bone Morphogenetic Protein 5
*FAF1*: Fas Associated Factor 1
GRHL3: Grainyhead Like Transcription Factor 3
CNC: Cranial neural crest
NCC: Neural crest cell
GWAS: Genome-wide association study
CNVs: Copy number variations
ChAS: Chromosomes analysis suite
CN: Copy number
STR: Short tandem repeats
ROH: Retention of heterozygosity
N1: Non-informative
UPD: Uniparental disomy
*TGFβ*: Transforming growth factor-beta
CHIP: Chromatin immunoprecipitation
